# Increased copy number for methylated maternal 15q duplications leads to changes in gene and protein expression in human cortical samples

**DOI:** 10.1186/2040-2392-2-19

**Published:** 2011-12-12

**Authors:** Haley A Scoles, Nora Urraca, Samuel W Chadwick, Lawrence T Reiter, Janine M LaSalle

**Affiliations:** 1 Medical Microbiology and Immunology, Genome Center, and Medical Institute of Neurodevelopmental Disorders, One Shields Avenue, University of California, Davis, Davis, CA 95616, USA; 2 Department of Neurology, University of Tennessee Health Science Center, 855 Monroe Ave., Link 415, Memphis, TN 38163, USA; 3 Department of Pediatrics, University of Tennessee Health Science Center, Memphis, TN 38163, USA

**Keywords:** autism, imprinting, copy number variation, 15q, duplication, methylation, epigenetic

## Abstract

**Background:**

Duplication of chromosome 15q11-q13 (dup15q) accounts for approximately 3% of autism cases. Chromosome 15q11-q13 contains imprinted genes necessary for normal mammalian neurodevelopment controlled by a differentially methylated imprinting center (imprinting center of the Prader-Willi locus, PWS-IC). Maternal dup15q occurs as both interstitial duplications and isodicentric chromosome 15. Overexpression of the maternally expressed gene *UBE3A *is predicted to be the primary cause of the autistic features associated with dup15q. Previous analysis of two postmortem dup15q frontal cortical samples showed heterogeneity between the two cases, with one showing levels of the GABA_A _receptor genes, *UBE3A *and *SNRPN *in a manner not predicted by copy number or parental imprint.

**Methods:**

Postmortem human brain tissue (Brodmann area 19, extrastriate visual cortex) was obtained from 8 dup15q, 10 idiopathic autism and 21 typical control tissue samples. Quantitative PCR was used to confirm duplication status. Quantitative RT-PCR and Western blot analyses were performed to measure 15q11-q13 transcript and protein levels, respectively. Methylation-sensitive high-resolution melting-curve analysis was performed on brain genomic DNA to identify the maternal:paternal ratio of methylation at PWS-IC.

**Results:**

Dup15q brain samples showed a higher level of PWS-IC methylation than control or autism samples, indicating that dup15q was maternal in origin. *UBE3A *transcript and protein levels were significantly higher than control and autism in dup15q, as expected, although levels were variable and lower than expected based on copy number in some samples. In contrast, this increase in copy number did not result in consistently increased *GABRB3 *transcript or protein levels for dup15q samples. Furthermore, *SNRPN *was expected to be unchanged in expression in dup15q because it is expressed from the single unmethylated paternal allele, yet *SNRPN *levels were significantly reduced in dup15q samples compared to controls. PWS-IC methylation positively correlated with *UBE3A *and *GABRB3 *levels but negatively correlated with *SNRPN *levels. Idiopathic autism samples exhibited significantly lower *GABRB3 *and significantly more variable *SNRPN *levels compared to controls.

**Conclusions:**

Although these results show that increased *UBE3A*/UBE3A is a consistent feature of dup15q syndrome, they also suggest that gene expression within 15q11-q13 is not based entirely on copy number but can be influenced by epigenetic mechanisms in brain.

## Background

Autism is a common neurodevelopmental disorder affecting 1 in 110 children [1], but its genetic etiology is complex and heterogeneous [2,3] Among the leading genetic causes of autism are abnormalities in proximal chromosome 15q, collectively referred to as "duplication 15 syndrome" (dup15q), which occur in ~1-3% of autism cases [4-6]. Dup15q syndrome is a clinically heterogeneous neurodevelopmental disorder characterized by varying degrees of cognitive impairment, gross motor delays, seizures, dysmorphic features and autism in 85% of cases [5,7,8].

Chromosome 15q11-q13 is a genetic hotbed for neurodevelopmental disorders because of a high density of low copy repeats (LCRs) that increase susceptibility to errors in meiotic recombination, resulting in deletions as well as duplications [9,10]. The parental origin of deletions and duplications influences the expression of 15q11-q13 through genomic imprinting. Imprinting of 15q11-q13 is regulated by the bipartite imprinting center (IC), which contains a differentially methylated region at the 5' end of *SNRPN *that controls expression throughout 15q11-q13 [11]. Loss of 15q11-q13 paternally expressed genes through deletions or maternal uniparental disomy (UPD) results in Prader-Willi syndrome (PWS), whereas the maternal deficiency at this locus results in a phenotypically distinct neurodevelopmental disorder, Angelman syndrome (AS).

Maternally derived duplications of chromosome 15, specifically 15q11-q13, are associated with an autistic phenotype, whereas paternally derived duplications primarily show normal phenotypes but may manifest neurological features other than autism [7,12]. In addition, individuals with PWS with maternal UPD show a higher occurrence of autism compared to those with PWS 15q11-q13 deletions [13,14], suggesting that overexpression at maternally expressed genes confers risk for autism [13,15]. The E3 ubiquitin ligase gene (*UBE3A*) is the only known paternally imprinted gene in the locus, and its maternal allele-specific transcription is limited to postnatal neurons [16,17]. While *ATP10A *was previously described as maternally expressed [18,19], recent studies have shown variable imprinting in humans and lack of imprinting in mouse [20,21]. Paternally expressed genes within 15q11-q13 include the splicing factor encoding *SNRPN*, necdin (*NDN*), *MAGEL2 *and several large clusters of small nucleolar RNAs (snoRNAs). A cluster of three receptor subunit genes for the neurotransmitter GABA_A _(*GABRB3, GABRA5, GABRG3*) are biallelically expressed in control brain tissue samples, but show epigenetic alterations that result in monoallelic expression in a subset of autism cortical samples [22].

Although increased copy number is generally assumed to increase transcript levels, the epigenetic and neurodevelopmental complexities associated with 15q11-q13 confound this simple explanation of *UBE3A *overexpression as the sole molecular cause of the dup15q phenotype. In addition to imprinting, the 15q11-q13 locus is subject to the interchromosomal higher organization of homologous pairing between maternal and paternal alleles [23-25]. Chromosome 15 duplications result in disrupted homologous pairing in dup15q brain tissue samples [26] and a neuronal cell line model of dup15q [24]. In addition, disruption of 15q11-q13 pairing by dup15q in neurons has been shown to result in reduced transcript levels of *NDN*, *SNRPN*, *GABRB3 *and *CHRNA7 *[24]. In a prior analysis of two dup15q cortical tissue samples, one showed reduced levels of the paternally derived transcripts *SNRPN*, snoRNAs, *NDN *and the biallelically expressed *GABRB3*, *GABRA5 *and *GABRG3 *transcripts that corresponded to PWS-like behaviors [26].

To further understand the genetic and epigenetic effects on transcript levels that lead to the pathogenesis of dup15q syndrome, we performed an extensive analysis of a panel of eight dup15q cortical tissue samples as compared to control and idiopathic autism samples. The dup15q samples showed changes in both methylation and transcription levels, with *UBE3A *showing significantly higher, *SNRPN *showing significantly lower and *GABRB3 *showing variable transcript levels as compared to controls. Interestingly, *UBE3A *transcript positively correlated with maternal allele-specific methylation of the Prader-Willi imprinting control locus (PWS-IC) within the dup15q samples. These results support the hypothesis that elevated UBE3A levels in the brain are a major contributor to the dup15q phenotype but also are consistent with observations that dup15q syndrome results in transcriptional and epigenetic changes that are variable and not based solely on copy number.

## Methods

### RNA extraction and cDNA synthesis

Frozen cerebral cortex samples from Brodmann area 19 (BA19) were obtained through the Autism Tissue Program from the University of Maryland Brain and Tissue Bank for Neurodevelopmental Disorders, the Harvard Brain and Tissue Resource Center and the University of Miami Brain and Tissue Bank for Neurodevelopmental Disorders. Brain tissues were stored at -80°C until processing. While the tissues were kept frozen on dry ice, 0.1 to 0.15 g was sliced and homogenized using TRIzol reagent (Invitrogen/Life Technologies, Carlsbad, CA, USA). All RNA work was done on a bench using pipettes wiped down with Ambion RNase*Zap *wipes (Invitrogen/Life Technologies, Austin, TX, USA) prior to beginning work to prevent RNase contamination of the sample. To eliminate DNA contamination, the RNA was treated with DNase I (New England Biolabs, Ipswich, MA, USA) according to the manufacturer's instructions and precipitated using sodium acetate and ethanol. Single-stranded cDNA was synthesized using QuantiTect Reverse Transcription Kit (QIAGEN, Valencia, CA, USA) and incubated for 15 minutes at 42°C followed by 15 minutes at 50°C with a 3-minute deactivation step at 95°C. For each reaction, a tube without RT was used as a control for genomic DNA contamination. After completion, each reaction was diluted 4.75-fold with DNase- and RNase-free water.

### Quantitative RT-PCR

Primers were designed using mRNA sequences extracted from the UCSC Genome Browser [27] with the February 2009 human reference sequence (GRCh37). Primers were designed to cross an intron or span intron-exon boundaries to limit genomic DNA contamination using Primer3 software [28] or Biosearch Technologies RealTimeDesign software (Biosearch Technologies Inc, Novato, CA, USA; http://www.biosearchtech.com/realtimedesign). These primer sequences are shown in Additional file 1. Each reaction was carried out in triplicate, and outliers were removed according to the method described by Bookout *et al. *[29]. PCR amplification of cDNA was performed using 200 nM primers and EXPRESS SYBR GreenER Universal Master Mix (Invitrogen/Life Technologies). Cycling conditions were 20 seconds at 95°C followed by 40 cycles of 2 seconds at 95°C and 30 seconds at 60°C. The reaction was performed using the Mastercycler ep *realplex *real-time PCR system (Eppendorf, Hamburg, Germany), and crossing points were analyzed using Realplex software. For each reaction, we ran a well of no RT and no cDNA control to evaluate genomic DNA contamination, nonspecific product formation or other contamination. Reaction conditions were as follows: 1× EXPRESS SYBR GreenER Universal Master Mix, 200 nM primers and 3.5 μl of cDNA. Cycling conditions were as follows: All samples were normalized to glyceraldehyde 3-phosphate dehydrogenase (*GAPDH*) using the comparative cycle threshold (C_T_) method (Applied Biosystems, Foster City, CA, USA) to measure fold changes relative to the calibrator, following normalization to *GAPDH*. Melting curve analysis was also performed to determine homogeneous product formation with no primer-dimers and no nonspecific products.

### Quantitative PCR for copy number analysis

Genomic DNA was isolated from brain tissue samples by using the Gentra Puregene Tissue Kit (QIAGEN). PCR conditions were the same as described above using primers shown in Additional file 1.

### Determination of methylation percentage at the imprinting center

Methylation-sensitive high-resolution melting-curve analysis (MS-HRM) was performed as described by Urraca *et al. *[30]. Briefly, 500 ng of DNA was treated with bisulfite. The treated sample (50 ng) was then used for MS-HRM on the LightCycler 480 Real-Time PCR System (Roche Applied Science, Indianapolis, IN, USA) using primers and conditions previously described [30]. Since the paternal *SNRPN *IC allele is completely unmethylated and the maternal allele is methylated, the normalized fluorescence intensity reveals the percentage of methylated (maternal) to unmethylated (paternal) allele present in a given sample. Additional copies of a maternal duplication of this region results in a higher percentage of maternal allele-specific (methylated) fluorescent signal.

### Protein level analyses

Protein extracts were isolated from the same frozen brain tissue samples using TRIzol reagent and analyzed for UBE3A and GABRB3 levels as described previously [31].

## Results

### Quantitative determination of genomic copy number and PWS-IC methylation in human brain samples

**Figure 1:**
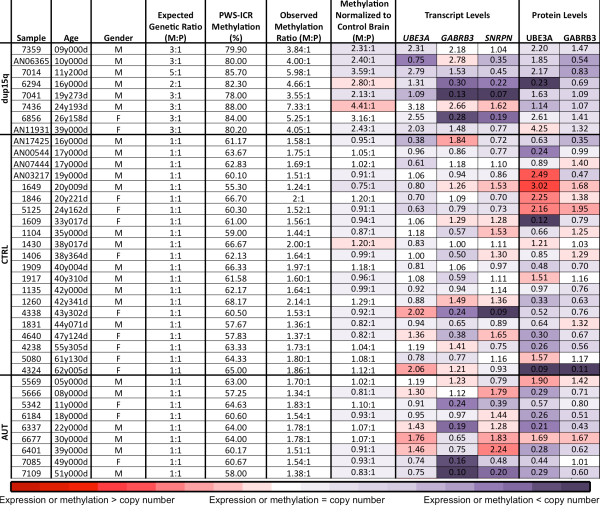
**Characteristics of individual postmortem human cortex tissue samples and experimental outcomes**. Age and gender are listed with the sample number (M, male; F, female). The expected genetic ratio is the number of maternal copies of 15q11-q13 to paternal copies (M:P). The percentage maternal allele-specific methylation in the fluorescent signal for both maternal and paternal melting peaks at the imprinting center of the Prader-Willi locus by methylation-sensitive high-resolution melting-curve analysis is shown in column 5 and then converted to a M:P ratio in column 6. Column 7 shows the M:P methylation ratio after normalization to control brain, with the average control (CTRL) set to 1.0. Columns 8 to 10 show the individual transcript levels normalized to CTRL brain. Protein levels are listed in columns 11 and 12. Each individual transcript and protein level was compared to the expected number of expressed copies relative to unaffected CTRL brain tissues. The data in columns 7 to 12 were then color-coded with a heat map used to visualize the fold changes as higher or lower than expected based on copy number and parental origin. Red is higher than and purple is lower than expected by copy number, with each intensity increase giving a 20% increase or decrease, respectively. *Case 7041 came from an individual who was on a respirator just prior to sample collection.

**Figure 2:**
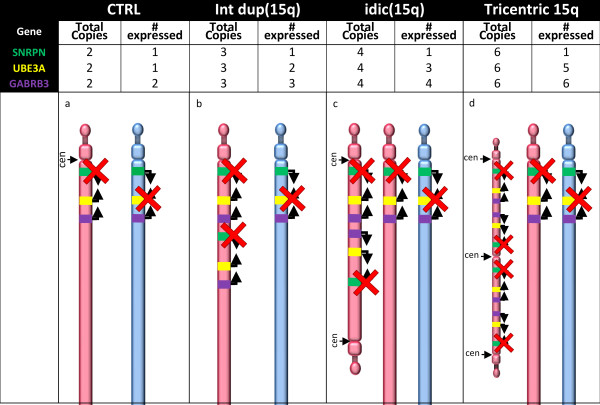
**Diagrams of chromosome 15q11-q13 gene order and expected expression patterns in duplication of 15q11-q13 (dup15q) and control samples based on copy number and parental origin**. The paternal chromosome is shown in blue and the maternal chromosome in red. Genes are listed and color blocks match, respectively, to model the location on the chromosome with the orientation indicated by arrows. Red × marks represent silencing from parental imprinting. The table at the top lists total genomic copies and the number of copies of each gene and color code: small nucleoriboprotein N (*SNRPN*), green; ubiquitin ligase 3A (*UBE3A*), yellow; and GABA_A _receptor β3 (*GABRB3*), purple. **(a) **Control (CTRL), **(b) **interstitial duplication of 15q (int dup(15q)) has one additional copy, **(c) **isodicentric duplication of chromosome 15 (idic(15q)) has two additional maternal copies of the locus and **(d) **the tricentric supernumerary chromosome 15, which contains four additional copies of chromosome 15.

To understand the relationship between increased maternal copy number of chromosome 15 and transcript levels in brain, frozen postmortem brain tissue samples from eight dup15q syndrome, twenty-one control and ten idiopathic autism human cortex tissue samples were obtained from BA19. Figure 1 documents the age, gender and expected 15q copy number extracted from Autism Tissue Program records. The chromosome 15 duplication and expected copy number were confirmed by quantitative PCR (qPCR) performed on genomic DNA from brain tissue samples at three loci (*SNRPN, UBE3A *and *GABRB3*) compared to an unduplicated control locus, *B2M*. Figure 2 shows the expected copy number and parental expression patterns of the three subtypes of dup15q in the collection of brain samples. Six of the dup15q samples showed twofold (2.10 ± 0.14, mean ± SEM) higher levels of 15q loci compared to control samples as expected for typical isodicentric 15q (idic15) duplications (Figure 2b). Case 6294 was confirmed as an interstitial 15q11-q13 duplication (int dup(15)) with 1.5-fold higher copy number as compared to controls with a total of three copies to each control's two (Figure 2c). Case 7014 has been described previously by both fluorescence *in situ *hybridization and array-comparative genomic hybridization as having a tricentric derivative of chromosome 15 [32], and qPCR confirmed a threefold excess of the three loci compared to controls (Figures 1 and 2d).

**Figure 3:**
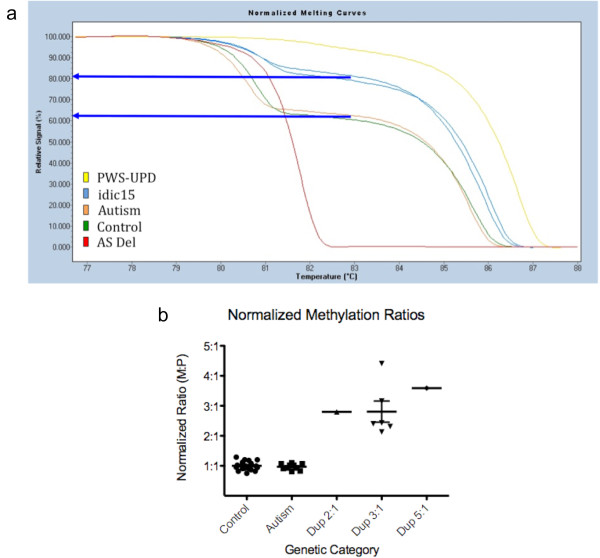
**The percentage of methylated imprinting center of the Prader-Willi locus (PWS-IC) shows a positive correlation with copy number at the 15q locus**. **(a) **Example of methylation-sensitive high-resolution melting-curve analysis showing > 80% methylated PWS-IC for duplication of 15q11-q13 (dup15q) sample 7041 (3:1 expected genetic ratio of the number of maternal copies of 15q11-q13 to paternal copies (M:P)) and sample 7014 (5:1 expected M:P ratio) (blue curves). All control and autism samples showed, on average, a 62% methylated PWS-IC (sample 6184 autism (orange) and sample 1649 control (green) here). The Prader-Willi syndrome uniparental disomy (PWS-UPD) (yellow) and the Angelman syndrome (AS) deletion (dark red) are shown as indicators of completely unmethylated (AS del) or completely methylated (PWS-UPD) signals from the PWS-IC. The blue arrows show the determination of percentage methylation from the percentage relative signal *y*-axis. **(b) **This graph presents the normalized methylation ratio (M:P) shown in Figure 1 grouped by genotype. Note that both control and autism samples cluster tightly at the 1:1 ratio in all samples. There was a single interstitial duplication sample (predicted 2:1 ratio) and a single complex isodicentric 15q (idic15) duplication sample (predicted 5:1 ratio), though the majority of cases examined were idic15q with four copies of the locus (predicted 3:1 ratio). Although somewhat variable, the mean ratio for the Dup 3:1 samples was approximately 3:1 and significantly different from both controls (*P *= 0.0037) and autism cases (*P *= 0.0035) by *t*-test using Welch's correction. The individual Dup 2:1 and Dup 5:1 samples showed higher and lower than predicted ratios, respectively. Error bars represent SEM. There was a positive correlation between the percentage methylation of the PWS-IC and the number of copies of the 15q region, which contains the PWS-IC, on the basis of simple regression analysis (*P *< 0.001).

Although maternally inherited of dup15q is much more common in autism cases, paternally derived duplications may also lead to phenotypic effects, including, but not exclusive to, autism [4]. Therefore, to verify the parental origin of the chromosome 15 duplication in each sample, analysis of the methylation status of the PWS-IC was performed by MS-HRM on the PCR product of bisulfite-converted DNA [30]. This method quantitates the percentage maternal vs paternal (that is, methylated DNA vs unmethylated DNA) at the PWS-IC. The ratio of methylated to unmethylated alleles is a clear indicator of the parental origin of the duplication because a higher methylated fluorescent signal means that more maternal (methylated) DNA than paternal DNA (unmethylated) is present, with the caveat that the 15q duplications have no IC defects, which could result in incomplete or inappropriate methylation of the *SNRPN *locus [30]. The ratio of maternal to paternal chromosomal copies (M:P) can be calculated by this method, given that the paternal PWS-IC is always unmethylated and the maternal PWS-IC is always methylated in normally developing controls (reviewed in [30]). Unexpectedly, this method revealed higher methylation levels in brain compared to blood samples that we previously analyzed at this locus, with an average of 61.7% (1.6:1 M:P ratio) in control brain compared to 52% previously observed in blood [30]. Brain tissue samples from idiopathic autism subjects exhibited methylation ratios similar to controls, with 61.3% methylation and a M:P ratio of 1.6:1. The observed methylation ratios were then "brain-normalized" to the average control maternal methylation percentage to give an average 1:1 M:P ratio for controls, which adjusted the average dup15q M:P ratio to 2.9:1 (Figure 1, column 7). Both observed and normalized methylation ratios were higher in all eight chromosome 15 duplication samples, confirming the maternal origin of the additional 15q alleles (Figure 3). There was some variation in the percentage methylation in the 3:1 expected ratio class, but this type of variation was also observed in the 2:1 ratio int dup(15) blood samples [30].

### Quantitation of UBE3A, SNRPN and GABRB3 transcript levels

To determine the effect dup15q and PWS-IC methylation may have on the transcript levels of the genes within this duplicated region, qRT-PCR was performed on RNA samples isolated from each brain tissue sample. Three chromosome 15 transcripts were amplified, including maternally expressed *UBE3A*, paternally expressed *SNRPN *and biallelically expressed *GABRB3 *(shown in Figure 2). The results were normalized to the chromosome 12 housekeeping control gene *GAPDH *by using the comparative C_T _method. The transcript levels, described as fold changes from average control, are shown for each brain tissue sample in Figure 1, columns 8 to 10. To visualize the direction and level of change from the expected transcript levels, heat map colors in Figure 1 were utilized, in which red is higher, purple is lower and white is < 20% change from expected.

**Figure 4:**
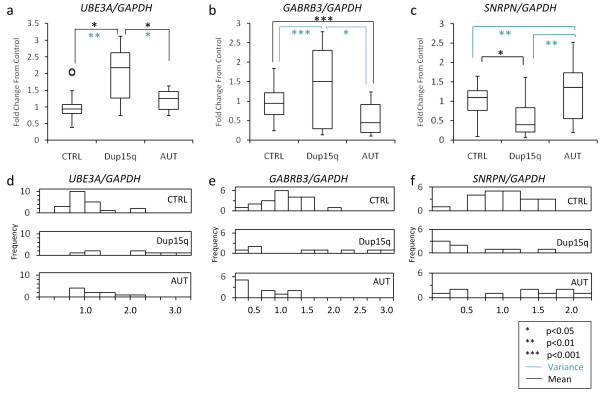
**Transcript level analyses of ubiquitin ligase 3A (*UBE3A*), GABA_A _receptor β3 (*GABRB3*) and small nucleoriboprotein N (*SNRPN*) in postmortem human brain tissue**. **(a) **through **(c) **box-and-whisker plots diagramming transcript levels in postmortem human brain normalized to glyceraldehyde 3-phosphate dehydrogenase (*GAPDH*). **(a) ***UBE3A *showed a significant increase in mean values for duplication of 15q11-q13 (dup15q) compared to control (CTRL) and autism (AUT). **(b) ***GABRB3 *was lower in AUT than in CTRL, whereas dup15q samples showed a significantly increased variance compared with both AUT and CTRL. **(c) ***SNRPN *expression levels were significantly lower in dup15q than in the other groups. In addition, AUT showed significantly increased variance for *SNRPN *levels compared to CTRL and dup15q. Significant differences are indicated by *P < 0.05, **P < 0.01 and ***P < 0.001. Significant differences in the variance between groups were determined by Levene's test for equality of variances, which are represented in blue. Black represents significant differences in the group means as determined by *t*-test. **(d) **through **(f) **Histograms representing the distribution of transcript levels in individual postmortem human brain grouped by condition are shown.

The imprinted gene *UBE3A *is expressed from the maternally derived duplication in addition to the normally inherited maternal chromosome. Idic15, int dup15 and the tricentric duplication would be expected to show 3×, 2× and 5× the number of expressed copies compared to both control and idiopathic autism, respectively (Figure 2). *UBE3A *transcript was significantly increased in dup15q samples compared to both the control group and autism (*P *= 0.004 and *P *= 0.045, respectively) (Figure 4a). In addition, the variability in *UBE3A *transcript levels between individual dup15q samples was significantly greater than in control or idiopathic autism samples (*P *= 0.002 and *P *= 0.045, respectively; Levene's test for equality of variances) (Figures 4a and 4d). When only samples with the same copy number (M:P ratio 3:1) were analyzed, *UBE3A *levels were an average twofold higher rather than the expected threefold (Additional file 2). When analyzed individually, five of the dup15q samples exhibited *UBE3A *levels higher than controls, whereas the other three dup15q samples showed levels similar to the control samples (Figure 4d). One idic15 sample (sample 7436) showed *UBE3A *transcript levels equivalent to the number of maternal copies (three) of *UBE3A *(Figure 1, column 8, and Figure 4d). However, the level of expression did not linearly correlate with the number of copies of the maternal allele present, with most dup15q samples showing lower *UBE3A *levels than expected (Figures 1 and 4d). These results indicate that elevated *UBE3A *levels are present in the brains of dup15q individuals, but that the levels are variable and generally lower than expected.

*GABRB3 *is biallelically expressed, so the idic15, int dup(15) and tricentric derivative chromosome M:P ratios were expected to be 2:1, 1.5:1 and 3:1, respectively, compared to control samples (Figure 2). *GABRB3 *levels in the dup15q cortex samples instead were highly variable, as three samples showed 0.3× lower expression and five samples showed 1.5× higher *GABRB3 *levels compared with typically developing controls (Figures 1, 4b and 4e). Figure 4b shows that the biallelically expressed *GABRB3 *exhibited no significant changes in the quantity of transcript in dup15q samples as a group; however, the variance in *GABRB3 *was significantly different from both the control group (*P *< 0.001) and the autism group (*P *< 0.001; *t*-test). Figure 4e of the distribution of *GABRB3 *levels shows two groups of dup15q samples clustered as either higher or lower than control samples. The idiopathic autism group showed a significant reduction in *GABRB3 *transcript compared with controls as reported previously [22].

*SNRPN *is paternally expressed and in all cases has one expressed copy, because all of the chromosome 15 duplications used were maternally derived. Surprisingly similar to an idic15 brain sample we previously reported from an individual with PWS-like features [26]. *SNRPN *was lower than expected by at least 0.77× in six of eight dup15q samples (Figures 1 and 4f) and significantly lower in dup15q samples compared to control or autism samples (Figure 4c). *SNRPN *levels were reduced from control in sample 6856 and increased from control in sample 7436, similar to findings of the previous study of BA9 prefrontal cortex [26]. Idiopathic autism showed no significant change in *SNRPN *level, but did show an increase in variability of the ten samples compared to control (*P *= 0.006; Levene's test for equality of variances) and to dup15q (*P *= 0.045; Levene's test for equality of variances).

### Correlation analyses of 15q transcript and protein levels with copy number and maternal PWS-IC methylation

**Figure 5:**
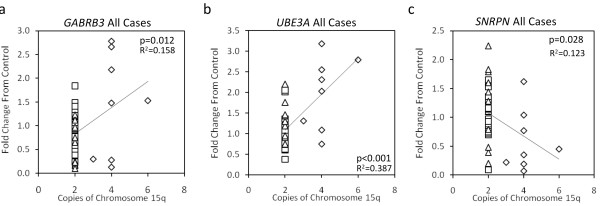
**Correlation analyses of 15q11-q13 copy number and transcript levels**. The relationship between the number of genomic copies of 15q11-q13 and transcript levels in all cases showed a predictive positive correlation with GABA_A _receptor β3 (*GABRB3*) **(a) **and ubiquitin ligase 3A (*UBE3A*) **(b)**, but a negative correlation with small nucleoriboprotein N (*SNRPN*) **(c)**. Significance was calculated by simple regression analysis. Diamond, duplication of 15q11-q13; triangle, autism; square, control.

As *UBE3A*, *GABRB3 *and *SNRPN *were expressed at lower levels than predicted by copy number and parental origin in dup15q brain, we sought to determine whether the transcript levels for genes within the dup15q locus correlate with the number of gene copies. *UBE3A *and *GABRB3 *would both be expected to show a positive correlation with the number of gene copies, because the duplication is maternally derived. A significant positive correlation in *GABRB3 *and *UBE3A *transcript levels was observed with increased chromosomal copies when all cases (control, autism and dup15q) were analyzed as a group (Figures 5a and 5b). This finding was not significant for the dup15q cases analyzed separately, although the positive trend was apparent (Additional file 3). *SNRPN *is paternally expressed and was therefore expected to show no correlation with the number of maternal copies of 15q; however, it showed a significant negative correlation of transcript with copy number for all cases (Figure 5c), but the correlation was not significant in the individually analyzed dup15q group (Additional file 3).

**Figure 6:**
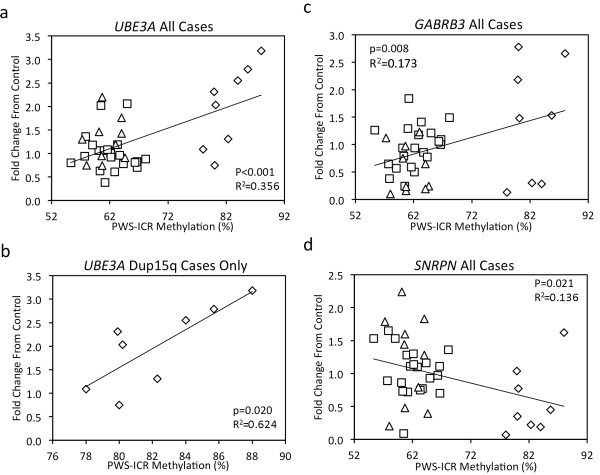
**Ubiquitin ligase 3A (*UBE3A*) and GABA_A _receptor β3 (*GABRB3*) levels positively correlate with imprinting center of the Prader-Willi locus (PWS-IC) methylation while small nucleoriboprotein N (*SNRPN*) levels negatively correlate with PWS-IC methylation**. A significant positive correlation between PWS-IC methylation and *UBE3A *transcript levels was observed when all cases were grouped together **(a) **or for duplication of 15q11-q13 (dup15q) samples only **(b)**. There was no significant correlation between percentage maternal allele-specific methylation at the PWS-IC and levels of *UBE3A *when cases were grouped separately without duplications as control or autism (Additional file 4). The percentage methylation of PWS-IC correlated with an increase in *GABRB3 *in all cases **(c)**. When separated by diagnosis, however, the positive trend between *GABRB3 *and PWS-IC methylation in dup15q, control, or autism cases analyzed separately did not reach significance (Additional file 5). A significant negative correlation between *SNRPN *and PWS-IC methylation was observed for all cases **(d)**; however, no significant correlation was observed for dup15q, control, or autism samples grouped separately for comparison of *SNRPN *and PWS-IC methylation (Additional file 6). Significance was calculated by a simple regression analysis. Diamond, dup15q; triangle, autism; square, control.

As expected on the basis of the maternal origin of the duplication, 15q11-q13 copy number correlated significantly with percentage PWS-IC methylation (Figure 3). *UBE3A *transcript level was significantly correlated with increased PWS-IC methylation for all samples (Figure 6a) and for the dup15q samples grouped separately (Figure 6b). When the control and autism cases were analyzed separately, however, no significant correlation between PWS-IC methylation and *UBE3A *was observed (Additional file 4). *GABRB3 *also significantly positively correlated with percentage PWS-IC methylation for all cases (Figure 6c). Unlike *UBE3A*, though, there was no significant correlation with percentage methylation in the dup15q cases grouped separately or with the control or autism cases alone (Additional file 5). In contrast to both *UBE3A *and *GABRB3*, *SNRPN *levels were significantly negatively correlated with percentage PWS-IC methylation in all cases (Figure 6d) but not with dup15q, control or autism cases analyzed separately (Additional file 6).

**Figure 7:**
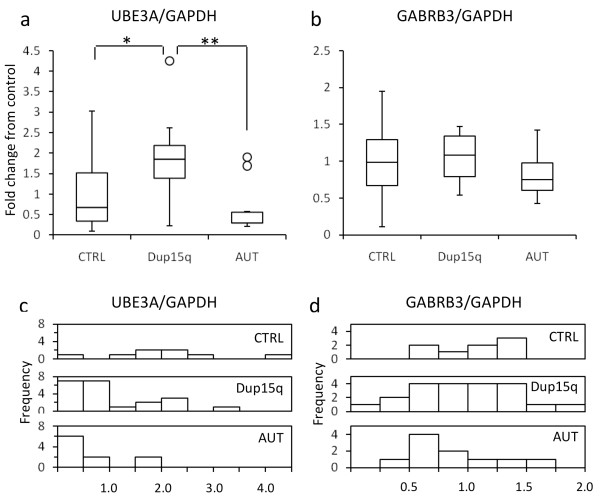
**Analysis of ubiquitin ligase 3A (UBE3A) and GABA_A _receptor β3 (GABRB3) protein levels in postmortem human brain samples**. Box-and-whisker plots diagramming protein levels in postmortem human brain tissue normalized to glyceraldehyde 3-phosphate dehydrogenase (GAPDH) by Western blot analysis with a significant increase of UBE3A protein level in duplication of 15q11-q13 (dup15q) **(a) **and no significant change in GABRB3 protein levels **(b)**. Significant differences are indicated by *P < 0.05 and **P < 0.01. Significant differences in the group means were determined by *t*-test. Distribution histograms of protein levels of individual brain tissue samples are shown in Additional file 7.

Protein was isolated from the same tissue as RNA and DNA from each brain sample to determine whether protein levels of 15q transcripts were significantly different between brain sample types or correlated with other measurements. Western blot analyses are only semiquantitative, thus, compared with qRT-PCR, we observed a larger variability between control samples (*P *= 0.02; Levene's test for equality of variances). Similarly to the transcript analyses, a significantly higher protein level of UBE3A was observed between control and dup15q (Figure 7a), but GABRB3 protein showed no significant differences between dup15q and control or autism samples (Figure 7b). No differences in variability within groups were observed at the GABRB3 protein level (Additional file 7), unlike our observations of *GABRB3 *transcript levels. At the protein level, both UBE3A and GABRB3 showed no significant relationship with copy number (Additional file 8) or percentage methylation of the PWS-IC (Additional files 9 and 10). The results therefore show that increased expression of both *UBE3A *transcript and UBE3A protein is a consistent feature of dup15q cortex.

## Discussion

This paper reports the largest study of dup15q brain samples to date. Our results demonstrate that duplication of the 15q11-q13 region alters the expression not only of *UBE3A*, as expected, but also the expression of *SNRPN *and *GABRB3 *in ways not always predicted by copy number, confirming our prior small-scale study [26]. Previously, *UBE3A *overexpression from the duplicated maternal allele had been hypothesized to be the sole explanation for autism comorbidity in dup15q syndrome as well as the increase in autism spectrum disorder (ASD) phenotypes in PWS maternal UPD compared to deletion cases [13,33]. It is important to keep in mind that the PWS-IC is methylated on all maternal alleles, regardless of allele copy number [34]. Even in studies of various nonneuronal cell lines, however, where *UBE3A *is expressed biallelically, increases in *UBE3A *transcript in the dup15q cells were observed [34-36]. Our study replicates the prior findings of increased *UBE3A *levels in human cortex, showing a twofold increase in dup15q samples. In contrast, *GABRB3 *expression was not analyzed in any of the prior studies in cell lines, because *GABRB3 *is a neuronally expressed gene. *SNRPN *is expressed in nonneuronal cell lines, but researchers in prior studies did not find *SNRPN *levels to be different from those of controls in nonneuronal cells [34-36]. In our investigation of dup15q human cortex samples, however, *SNRPN *levels were significantly lower than in controls, a result that we did not expect, since all of the samples (control, autism and dup15) should express one copy of the *SNRPN *gene from the single paternal allele present. Our results therefore demonstrate the tissue-specific epigenetic complexities associated with dup15q syndrome in humans which simple copy number changes are inadequate to explain.

Epigenetic patterns and mechanisms are often tissue-specific, and the brain shows high levels of DNA methylation despite being primarily nonmitotic in postnatal life [37]. Our recent genomic analysis of DNA methylation showed large genomic regions that are highly methylated in neurons compared to fibroblasts that span large regions of 15q11-q13 [38]. Interestingly, in this study, we observed tissue-specific differences in PWS-IC methylation between brain tissues as compared to blood samples analyzed previously [30] by MS-HRM, with brain tissue showing a higher percentage of baseline maternal allele-specific methylation in controls. The MS-HRM analysis of the PWS-IC upstream of *SNRPN *showed that, when normalized to brain, a M:P methylation ratio of 2.9:1 was observed, indicating that the duplications are maternal in origin. The increased methylation observed in dup15q samples is consistent with findings of previous studies in blood from int dup(15) samples showing that the duplication is maternal, not paternal, in origin. However, it is possible that the paternal allele may be methylated at one or more individual bases in the dup15q samples only. The recent discovery of 5-hydroxymethylcytosine (5-hmC) [39,40] may be of significance in this regard, because more 5-hmC has been found in brain than in other tissues [41] and 5-hmC is thought to affect gene regulation through DNA demethylation [42] or by converting 5-methylcytosine (5-mC) to 5-hmC [43-45]. Further investigation of the methylation status of the PWS-IC in brain samples is needed to determine whether the bisulfite-converted sites are protected by 5-hmC or 5-mC.

*UBE3A *transcript and protein levels were increased twofold on average in dup15q samples compared to controls in our study, consistent with the hypothesis that there is increased maternal allele-specific expression of *UBE3A *in dup15q autism brain. These levels were slightly lower than expected from maternally expressed genes with an average of three maternal alleles, but this may reflect the complex transcriptional and posttranslational regulation of *UBE3A*. The function of UBE3A as a transcriptional coactivator has been largely unexplored in the context of human genetic disease, but, in a *Drosophila *model of 15q duplication syndrome, elevated levels of an enzymatically defective version of Dube3a were able to induce transcription of the dopamine regulator GTP cyclohydrolase I and elevate dopamine levels in the fly brain [46]. UBE3A can *trans*-ubiquitinate itself *in vivo*, leading to self-degradation, supporting the idea that there is an upper limit for UBE3A protein induction that may be reached in as few as two active copies of the duplicated region.

Dup15q sample 6,856 showed a 2.5-fold increase in *UBE3A *compared to no significant change as seen previously for a different brain region from this individual [26]. Brain region differences in transcript levels within the same individual may explain some of the clinical heterogeneity seen within the dup15q syndrome. They may also potentially be explained by the stochastic nature of the epigenetic dysregulation. Interestingly, the epigenetic measure that best correlated with *UBE3A *levels in the dup15q brain samples was the level of PWS-IC methylation. Since the correlation was positive rather than negative, we hypothesize that maternal PWS-IC methylation acts as a long-range enhancer of *UBE3A *expression. The methyl-binding protein MeCP2 binds to the methylated PWS-IC allele [25,31,47,48], and *MECP2 *mutation has been shown to correspond with reduced UBE3A and GABRB3 levels in human brain [31]. Therefore, increased binding of MeCP2 to highly methylated PWS-IC in brain may act as a positive transcriptional regulator of *UBE3A *and, to a lesser extent, *GABRB3 *in human cortex.

In contrast to *UBE3A*, *GABRB3 *exhibited no significant change in the mean expression in the dup15q cortical samples compared to controls. Instead, significant variability in *GABRB3 *levels, as well as an interesting bimodal separation in *GABRB3 *levels of the dup15q samples, was observed in dup15q samples. This result is similar to our findings in a prior study of two samples with discordant *GABRB3 *levels [26], as well as the finding of reduced GABRB3 levels in 56% of autism cortex samples [22]. *SNRPN *levels were decreased overall in all dup15q samples in this study, which deviates from our previous study result from sample 7014 at a different brain region, BA9, examined previously [26]. This unexpected result of reduced *SNRPN *in dup15q postmortem cortex samples suggests that an increase in maternal dosage of the region epigenetically affects transcription of a paternally expressed gene, possibly in a tissue- or region-specific manner. In contrast to *UBE3A *and *GABRB3*, which positively correlated with PWS-IC methylation, *SNRPN *levels showed a negative correlation with PWS-IC methylation. These results suggest that although maternal methylation of the PWS-IC is repressive to *SNRPN *expression, as expected, there appears to be a long-range enhancing effect of PWS-IC methylation on *UBE3A *and *GABRB3*. Homologous chromosome pairing of maternal and paternal 15q11-q13 alleles occurs in human lymphocytes, neuronal cells and brain [23-25]. Both dup15q brain samples and a neuronal cell culture model of dup15q in SH-SY5Y neuronal cells showed significant disruption of homologous pairing that corresponded to reduced *SNRPN *and lower than expected *GABRB3 *levels [24,26].

## Conclusions

This study, together with previous studies of dup15q syndrome, shows that dup15q brain samples are epigenetically complex and that 15q11-q13 transcripts in brain do not behave solely as predicted by copy number. These findings should be important for understanding ASD cases with other *de novo *copy number variations on other chromosomes, in particular large duplications [49]. The bimodal pattern of *GABRB3 *deficiencies seen in these 8 dup15q samples may provide some insight into the relationship between dup15q and seizures. Maternal UPD PWS individuals have a higher incidence of seizures than individuals with deletions [14], suggesting that these people may also have epigenetically induced *GABRB3 *deficiency. *GABRB3 *and *UBE3A *are well-characterized candidate genes for ASD because they are associated with normal brain development and have been shown to be reduced in idiopathic autism, Angelman syndrome and Rett syndrome [31]. Although these results provide support for the hypothesis that overexpression of the maternally expressed *UBE3A *gene in the brain is the primary underlying cause of the ASD phenotype in dup15q, the changes in *GABRB3 *and *SNRPN *expression not predicted by copy number may also influence the phenotypic variability observed in ASD.

## Abbreviations

AS: Angelman syndrome; dup15q: duplication of 15q11-q13; IC: imprinting control locus; idic15: isodicentric 15q; int dup(15): interstitial duplication 15q; GABA: γ-aminobutyric acid; GABRB3: GABA_A _receptor β3; GAPDH: glyceraldehyde 3-phosphate dehydrogenase; LCR: low copy repeat; MS-HRM: methylation-sensitive high-resolution melting-curve analysis; PWS: Prader-Willi syndrome; PWS-IC: imprinting center of the Prader-Willi locus; RT-PCR: reverse transcriptase polymerase chain reaction; snoRNA: small nucleolar RNA; SNRPN: small nucleoriboprotein N; UBE3A: ubiquitin ligase 3A; UPD: uniparental disomy.

## Competing interests

The authors declare that they have no competing interests.

## Authors' contributions

HAS carried out the molecular genetic studies and drafted the manuscript. NU carried out the MS-HRM analyses. SWC carried out protein/RNA isolations and Western blot analyses. LTR participated in the study design and coordination and helped to draft the manuscript. JML conceived the study, participated in its design and coordination and helped to draft the manuscript. All authors read and approved the final manuscript.

## Supplementary Material

Additional file 1 **Primers used in this study**. Primer sequences used for quantitative RT-PCR and copy number analyses are provided. Click here for file

Additional file 2 **Ubiquitin ligase 3A (*UBE3A*) transcript levels are significantly higher in duplication of 15q11-q13 (dup15q) copy number samples than in control and autism brain tissues**. Fold change vs genotype for *UBE3A *levels in brain tissue. Click here for file

Additional file 3 **Correlation analyses of 15q11-q13 copy number and transcript levels**. This analysis was performed as explained in Figure 5, except that only the duplication of 15q11-q13 (dup15q) samples were correlated with copy number. Click here for file

Additional file 4 **Correlation analyses of imprinting center of the Prader-Willi locus (PWS-IC) methylation and ubiquitin ligase 3A (*UBE3A*) transcript levels**. This analysis was performed as explained in Figures 6a and 6b, except that only the controls or autism samples were correlated with PWS-IC methylation. Click here for file

Additional file 5 **Correlation analyses of imprinting center of the Prader-Willi locus (PWS-IC) and GABA_A _receptor β3 (*GABRB3*) transcript levels**. This analysis was performed as explained in Figure 6c, except that only the duplication of 15q11-q13 (dup15q), controls or autism samples were correlated with PWS-IC methylation. Click here for file

Additional file 6 **Correlation analyses of imprinting center of the Prader-Willi locus (PWS-IC) methylation and *SNRPN *transcript levels**. This analysis was performed as explained in Figure 6d, except that only the duplication of 15q11-q13 (dup15q), controls or autism samples were correlated with PWS-IC methylation. Click here for file

Additional file 7 **Distribution of ubiquitin ligase 3A (UBE3A) and GABA_A _receptor β3 (GABRB3) protein levels in individual brain samples by condition**. Western blot analyses of protein levels were performed as described in Figure 7 for UBE3A or GABRB3. Click here for file

Additional file 8 **Protein levels showed no significant association with copy number**. We found no significant relationship between UBE3A or GABRB3 protein levels and copy number in all cases or in duplication of 15q11-q13 (dup15q) only. Click here for file

Additional file 9 **Ubiquitin ligase 3A (UBE3A) protein levels did not correlate with imprinting center of the Prader-Willi locus (PWS-IC) methylation**. In all cases, the positive trend between UBE3A protein level and methylation was similar to transcript level and methylation; however, it did not reach significance, nor did the other groups when analyzed separately. Click here for file

Additional file 10 **GABA_A _receptor β3 (GABRB3) protein levels did not correlate with imprinting center of the Prader-Willi locus (PWS-IC) methylation**. When all three groups were analyzed together or separately, there was no correlation between percentage PWS-IC methylation and GABRB3 protein levels. Click here for file
