# Supplementary material for: Increased copy number for methylated maternal 15q duplications leads to changes in gene and protein expression in human cortical samples
Source: Mol Autism. 2011 Dec 12;2:19. doi: 10.1186/2040-2392-2-19 (PMC3287113; doi:10.1186/2040-2392-2-19)
Supplement: Additional file 1 — Primers used in this study. Primer sequences used for quantitative RT-PCR and copy number analyses are provided. [file 2040-2392-2-19-S1.PDF]

Primers Employed for qRT-PCR

Gene name	Forward primer	Reverse primer	Product size (bp)
**Human:**			
CHRNA7	GACGTGGATGAGAAGAACCAA	TGGGAAACGAACAGTCTTCAC	120
GABRB3	ACTCCGGTAACAGCCTTGT	CAGAACTGCACTCTGGAAAT	92
GAPDH	TGAACCATGAGAAGTATGACAAC	GTCCTTCCACGATACCAAAG	116
SNRPN	GGACTCCCATCAAGTCTCCA	AAAAGGGGCAGCAAAAATCT	139
UBE3A	CCATGGGAAAATGTACATCCA	TTTTTCAGCTGGTTGTGGAGG	186

Copy Number Primers:

Gene name	Forward primer	Reverse primer	Product size (bp)
UBE3A	TCACGAATGTGCTCAGAAACT	TGGCCCAGACACAGAAAG	118
GABRB3	GGGTGGTGCCTCTACCTACA	TGCCTCACCCTCTCTGTTCT	91
**CTRL (β2 microglobulin)***	AGACAAGTCTGAATGCTCCACTTTT	TGGGTTTCATCCATCCGACATT	82
SNRPN	CGGAAATCCCTTACTCCA	TTGATCCTCTGTGATTGTGA	117

* [18]
