# Supplementary material for: Increased copy number for methylated maternal 15q duplications leads to changes in gene and protein expression in human cortical samples
Source: Mol Autism. 2011 Dec 12;2:19. doi: 10.1186/2040-2392-2-19 (PMC3287113; doi:10.1186/2040-2392-2-19)
Supplement: Additional file 2 — Ubiquitin ligase 3A (UBE3A) transcript levels are significantly higher in duplication of 15q11-q13 (dup15q) copy number samples than in control and autism brain tissues. Fold change vs genotype for UBE3A levels in brain tissue. [file 2040-2392-2-19-S2.PDF]

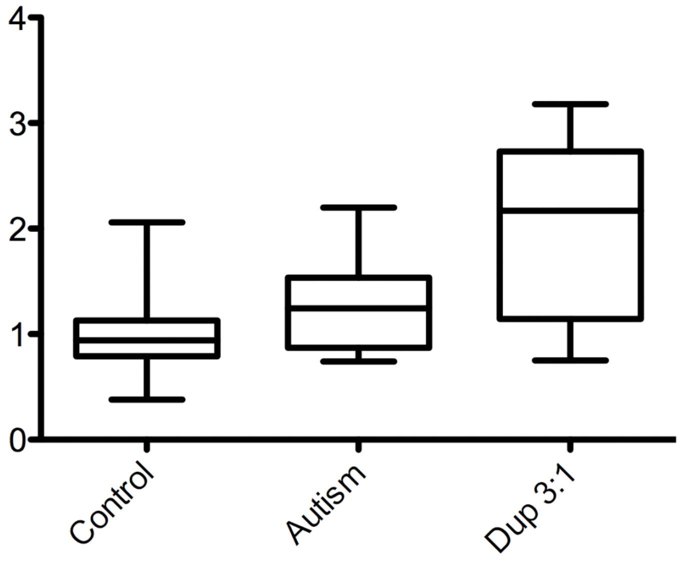

***UBE3A* transcript levels are significantly higher in Dup15q 3:1 copy number samples as compared to control and autism brain.** Fold change vs. genotype for *UBE3A* levels in brain. Although somewhat variable, the duplication samples with 3:1 duplication ratios were significantly higher than the control group (*t*-test p=0.0148 for control with Welch's correction) but not the autism group. The whisker bars are maximum and minimum values in each group and the middle bar is the mean expression value.
