# Supplementary material for: Increased copy number for methylated maternal 15q duplications leads to changes in gene and protein expression in human cortical samples
Source: Mol Autism. 2011 Dec 12;2:19. doi: 10.1186/2040-2392-2-19 (PMC3287113; doi:10.1186/2040-2392-2-19)
Supplement: Additional file 3 — Correlation analyses of 15q11-q13 copy number and transcript levels. This analysis was performed as explained in Figure 5, except that only the duplication of 15q11-q13 (dup15q) samples were correlated with copy number. [file 2040-2392-2-19-S3.PDF]

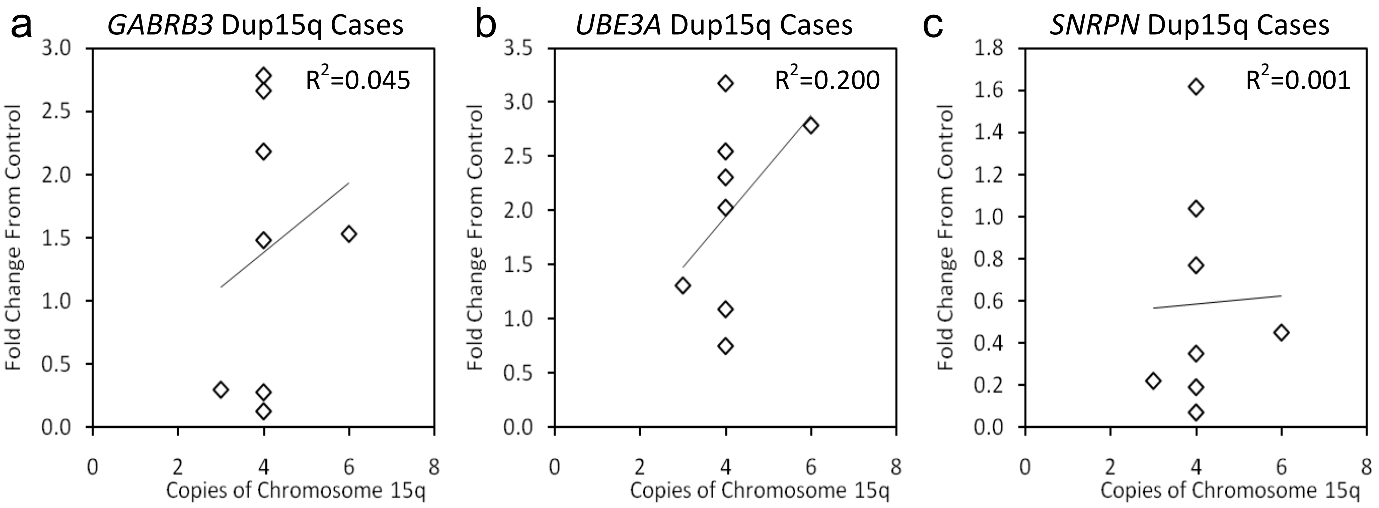

Correlation analyses of 15q11-q13 copy number and transcript levels. The analysis was performed as explained in Figure 5 except only the Dup15q samples were correlated with copy number. None of the correlations were significant when the dup15q samples were separately analyzed (a-c), although postitive trends were observed for *GABRB3* and *UBE3A* (a, b), but not *SNRPN* (c). Significance was calculated by a simple regression analysis.
