# Supplementary material for: Increased copy number for methylated maternal 15q duplications leads to changes in gene and protein expression in human cortical samples
Source: Mol Autism. 2011 Dec 12;2:19. doi: 10.1186/2040-2392-2-19 (PMC3287113; doi:10.1186/2040-2392-2-19)
Supplement: Additional file 4 — Correlation analyses of imprinting center of the Prader-Willi locus (PWS-IC) methylation and ubiquitin ligase 3A (UBE3A) transcript levels. This analysis was performed as explained in Figures 6a and 6b, except that only the controls or autism samples were correlated with PWS-IC methylation. [file 2040-2392-2-19-S4.PDF]

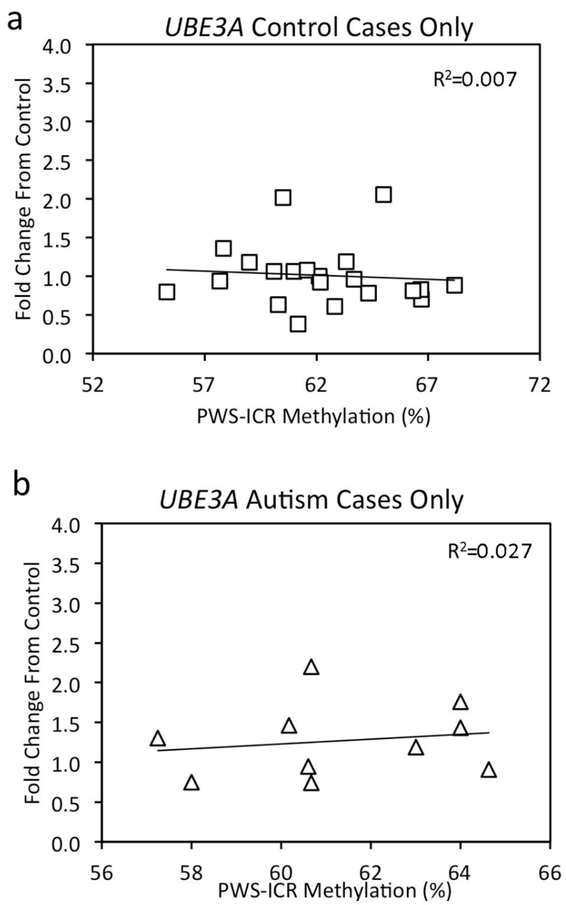

Correlation analyses of PWS-IC methylation and *UBE3A* transcript levels. The analysis was performed as explained in Figure 6a-b except only the controls (a) or autism (b) samples were correlated with PWS-ICR methylation. There was no significant correlation between percent maternal allele specific methylation at the PWS-IC and levels of *UBE3A* when separately grouping the cases without duplications as control or autism. Significance was calculated by a simple regression analysis.
