# Supplementary material for: Increased copy number for methylated maternal 15q duplications leads to changes in gene and protein expression in human cortical samples
Source: Mol Autism. 2011 Dec 12;2:19. doi: 10.1186/2040-2392-2-19 (PMC3287113; doi:10.1186/2040-2392-2-19)
Supplement: Additional file 5 — Correlation analyses of imprinting center of the Prader-Willi locus (PWS-IC) and GABAA receptor β3 (GABRB3) transcript levels. This analysis was performed as explained in Figure 6c, except that only the duplication of 15q11-q13 (dup15q), controls or autism samples were correlated with PWS-IC methylation. [file 2040-2392-2-19-S5.PDF]

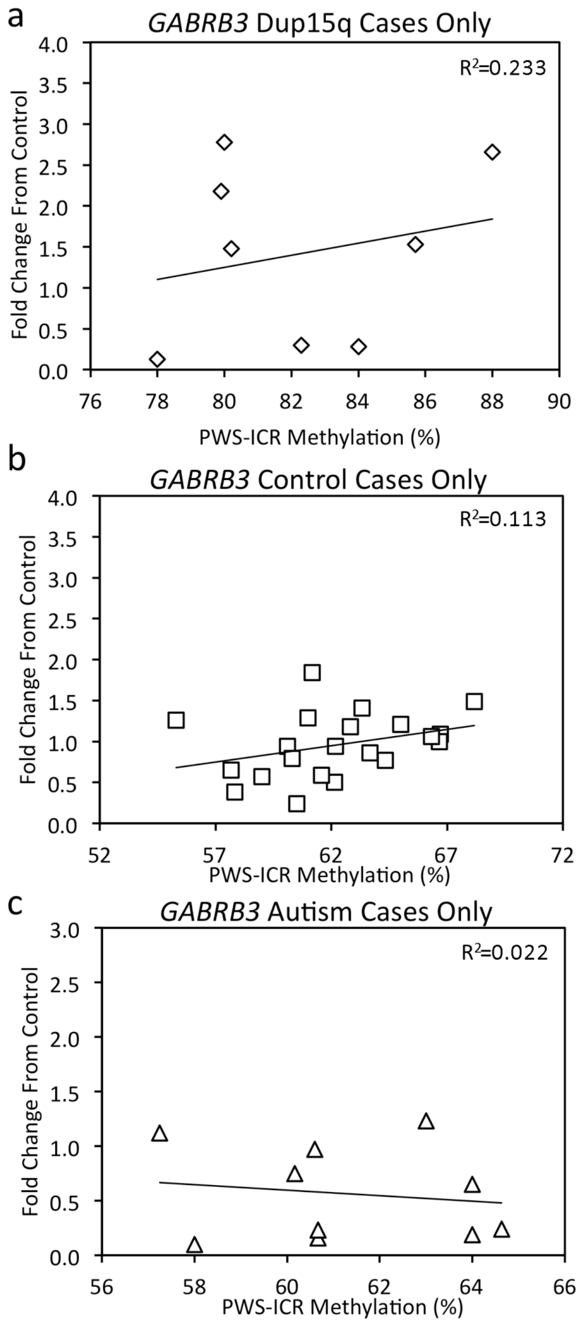


**Correlation analyses of PWS-IC methylation and *GABRB3* transcript levels.** The analysis was performed as explained in Figure 6c except only the Dup15q (a) controls (b) or autism (c) samples were correlated with PWS-ICR methylation. There was no significant correlation between percent maternal allele specific methylation at the PWS-IC and levels of *GABRB3* when separately grouping the cases. Significance was calculated by a simple regression analysis.
