# Supplementary material for: Increased copy number for methylated maternal 15q duplications leads to changes in gene and protein expression in human cortical samples
Source: Mol Autism. 2011 Dec 12;2:19. doi: 10.1186/2040-2392-2-19 (PMC3287113; doi:10.1186/2040-2392-2-19)
Supplement: Additional file 7 — Distribution of ubiquitin ligase 3A (UBE3A) and GABAA receptor β3 (GABRB3) protein levels in individual brain samples by condition. Western blot analyses of protein levels were performed as described in Figure 7 for UBE3A or GABRB3. [file 2040-2392-2-19-S7.PDF]

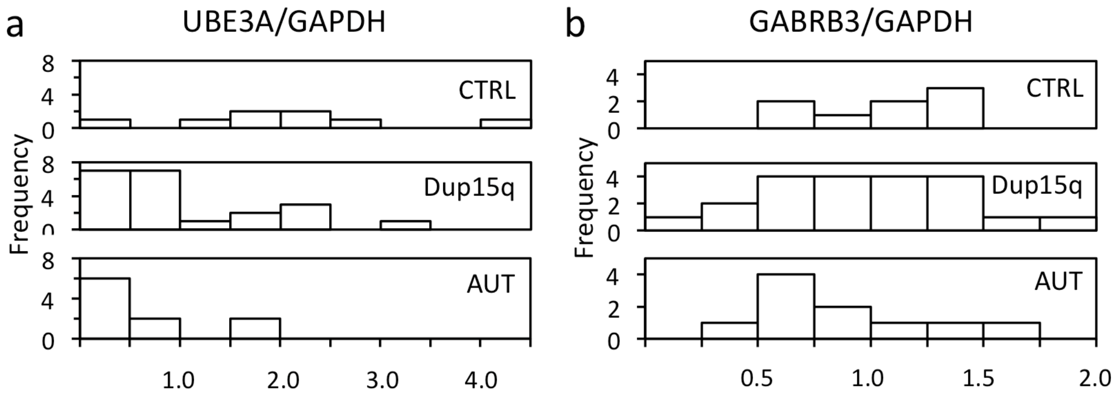


**Distribution of UBE3A and GABRB3 protein levels in individual brain samples by condition.** Western blot analyses of protein levels were performed as in Figure 7 for UBE3A (a) or GABRB3 (b). Histograms represent the distribution of protein levels in postmortem human brain separated by condition.
