# Supplementary material for: Increased copy number for methylated maternal 15q duplications leads to changes in gene and protein expression in human cortical samples
Source: Mol Autism. 2011 Dec 12;2:19. doi: 10.1186/2040-2392-2-19 (PMC3287113; doi:10.1186/2040-2392-2-19)
Supplement: Additional file 9 — Ubiquitin ligase 3A (UBE3A) protein levels did not correlate with imprinting center of the Prader-Willi locus (PWS-IC) methylation. In all cases, the positive trend between UBE3A protein level and methylation was similar to transcript level and methylation; however, it did not reach significance, nor did the other groups when analyzed separately. [file 2040-2392-2-19-S9.PDF]

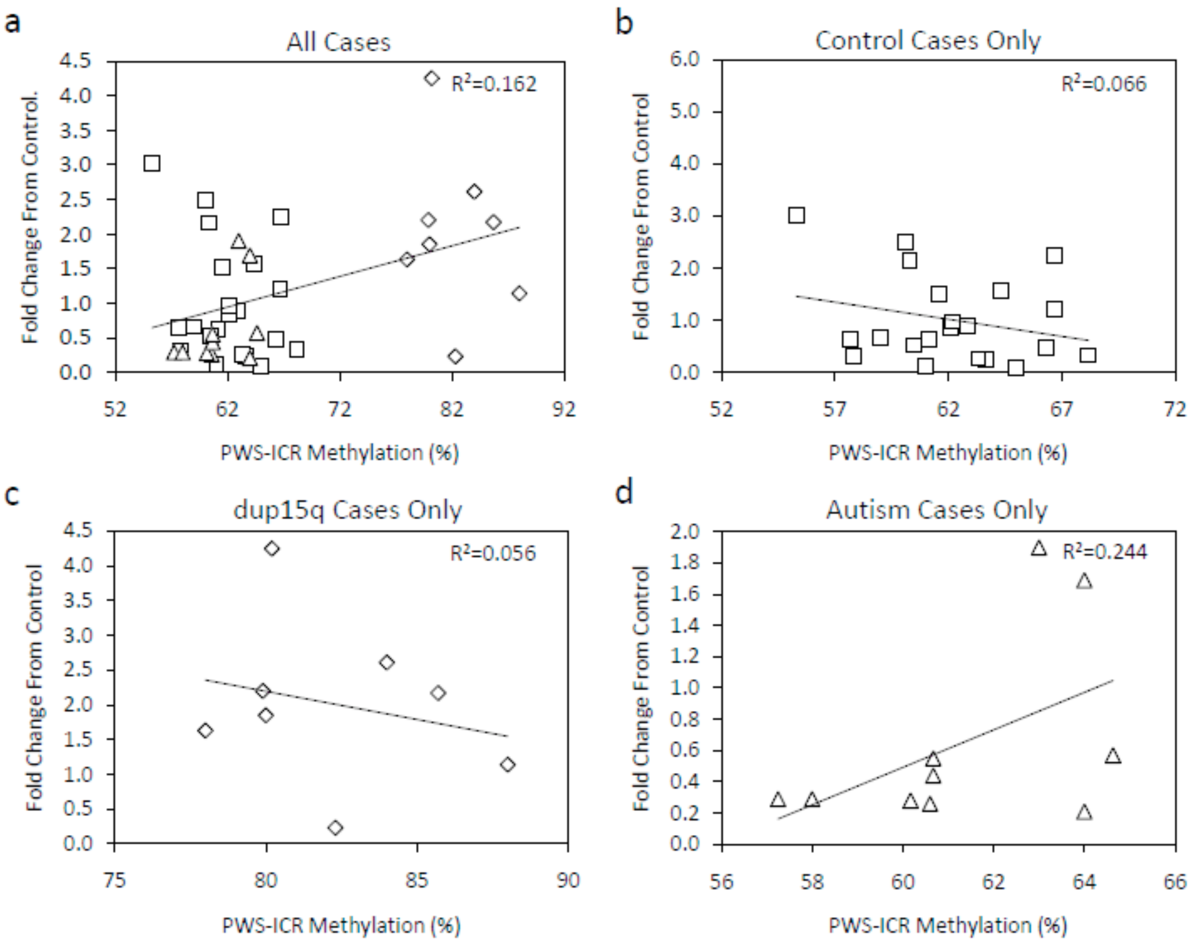

UBE3A protein levels did not correlate with PWS-IC methylation.

In all cases the positive trend between UBE3A protein level and methylation was similar to transcript level and methylation however it did not reach significance (a) nor did the other groups when analyzed separately (b-d). Significance was calculated by a simple regression analysis. (◊ Dup15q, Δ Autism, □ Control)
