# Supplementary material for: Increased copy number for methylated maternal 15q duplications leads to changes in gene and protein expression in human cortical samples
Source: Mol Autism. 2011 Dec 12;2:19. doi: 10.1186/2040-2392-2-19 (PMC3287113; doi:10.1186/2040-2392-2-19)
Supplement: Additional file 10 — GABAA receptor β3 (GABRB3) protein levels did not correlate with imprinting center of the Prader-Willi locus (PWS-IC) methylation. When all three groups were analyzed together or separately, there was no correlation between percentage PWS-IC methylation and GABRB3 protein levels. [file 2040-2392-2-19-S10.PDF]

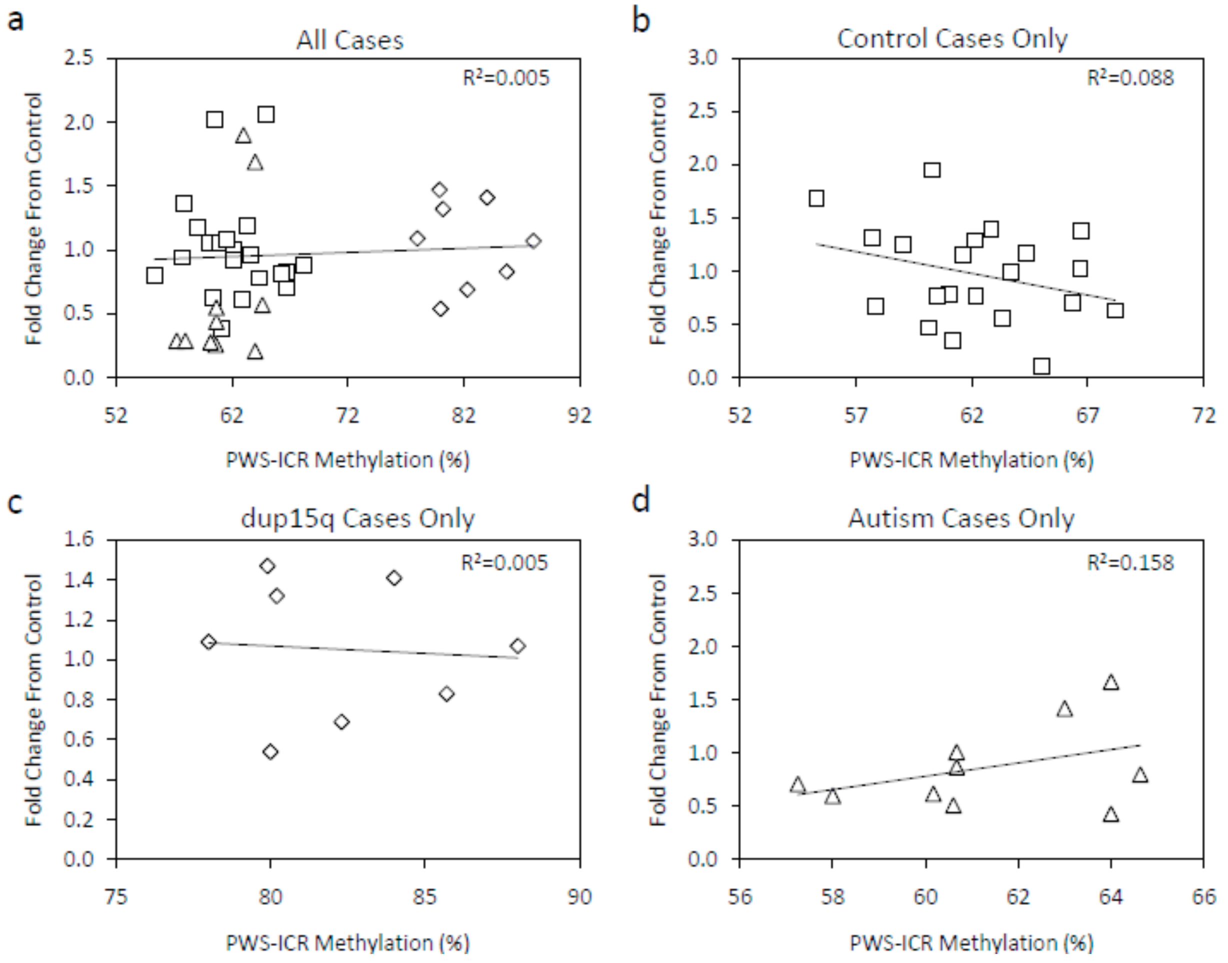

GABRB3 protein levels did not correlate with PWS-IC methylation. When all three groups were analyzed together or separately there was no correlation between % PWS-IC methylation and GABRB3 protein levels.
