# Supplementary material for: Increased copy number for methylated maternal 15q duplications leads to changes in gene and protein expression in human cortical samples
Source: Mol Autism. 2011 Dec 12;2:19. doi: 10.1186/2040-2392-2-19 (PMC3287113; doi:10.1186/2040-2392-2-19)
Supplement: Additional file 8 — Protein levels showed no significant association with copy number. We found no significant relationship between UBE3A or GABRB3 protein levels and copy number in all cases or in duplication of 15q11-q13 (dup15q) only. [file 2040-2392-2-19-S8.PDF]

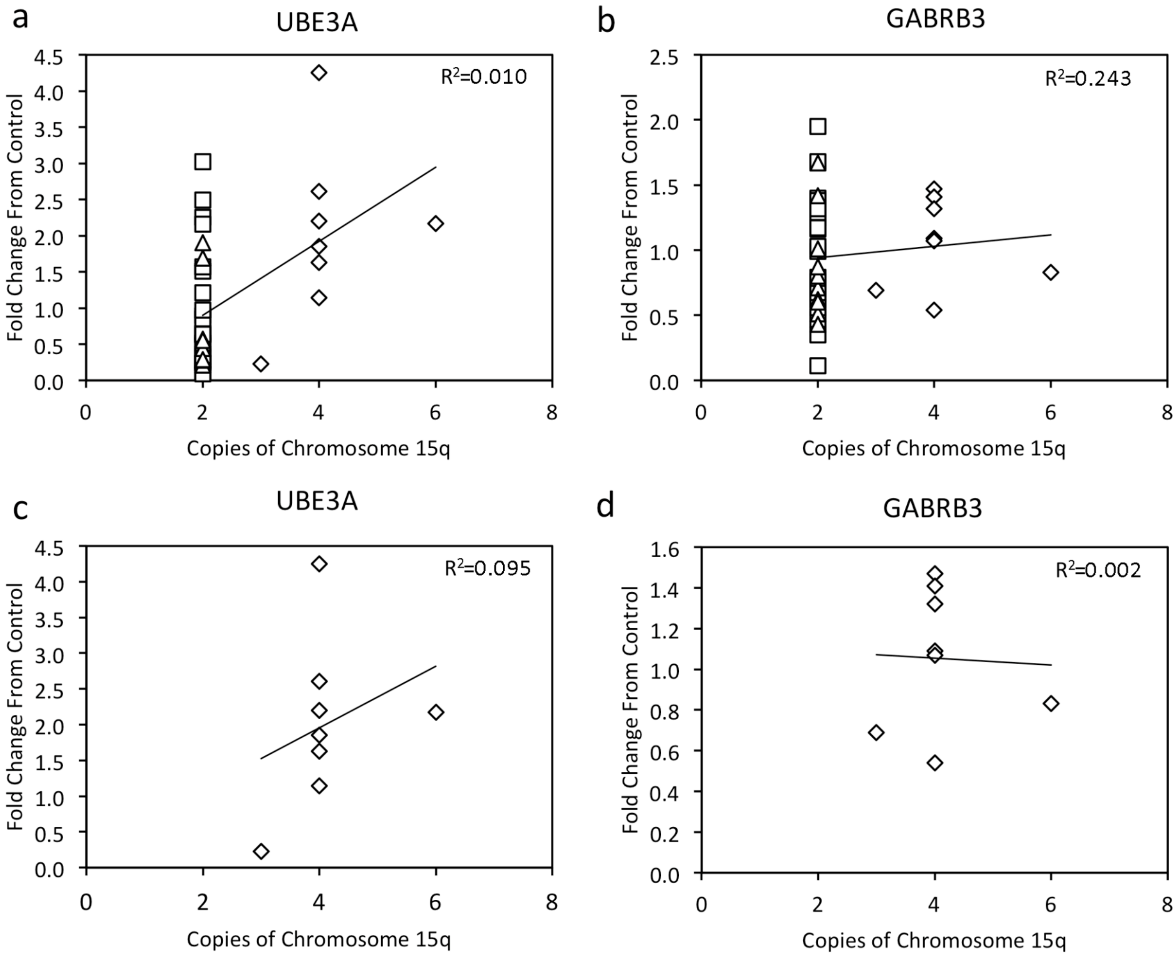

Protein levels showed no significant association with copy number. There was no significant relationship between UBE3A or GABRB3 protein levels in all cases (a-b) or in Dup15q only (c-d). Significance was calculated by a simple regression analysis. (◊ Dup15q, Δ Autism, □ Control).
